# The impact of rheumatologist-performed ultrasound on diagnosis and management of inflammatory arthritis in routine clinical practice

**DOI:** 10.1186/s12891-017-1850-4

**Published:** 2017-11-22

**Authors:** Stephen Kelly, Brian Davidson, Sarah Keidel, Stephan Gadola, Claire Gorman, Gary Meenagh, Piero Reynolds

**Affiliations:** 1 Barts Health NHS Trust, London, UK; 2 University Hospital Southampton NHS Foundation Trust, Southampton, UK; 3 Abbvie Limited, Maidenhead, UK; 4 Homerton University Hospital NHS Foundation Trust, London, UK; 5 Antrim Area Hospital, Antrim, Northern Ireland UK

**Keywords:** Arthritis, Ultrasound, DMARD

## Abstract

**Background:**

Rheumatologists increasingly perform ultrasound (US) imaging to aid diagnosis and management decisions. There is a need to determine the role of US in facilitating early diagnosis of inflammatory arthritis. This study describes the impact of US use by rheumatologists on diagnosis and management of inflammatory arthritis in routine UK clinical practice.

**Methods:**

We conducted a prospective study in four secondary care rheumatology clinics, each with one consultant who routinely used US and one who did not. Consenting patients aged > 18, newly referred with suspected inflammatory arthritis were included. Data were collected both retrospectively from medical records and via a prospectively-completed physician questionnaire on US use. Analyses were stratified by US/non-US groups and by sub-population of rheumatoid arthritis (RA)-diagnosed patients.

**Results:**

258 patients were included; 134 US and 124 non-US. 42% (56/134) of US and 47% (58/124) of non-US were diagnosed with RA. Results described for US and non-US cohorts, respectively as follows. The proportion of patients diagnosed at their first clinic visit was 37% vs 19% overall (*p* = 0.004) and 41% vs 19% in RA-diagnosed patients (*p* = 0.01). The median time to diagnosis (months) was 0.85 vs 2.00 (overall, *p* = 0.0046) and 0.23 vs 1.38 (RA-diagnosed, *p* = 0.0016). Median time (months) to initiation on a DMARD (where initiated) was 0.62 vs 1.41 (overall, *p* = 0.0048) and 0.46 vs 1.81 (RA-diagnosed, *p* = 0.0007).

**Conclusion:**

In patients with suspected inflammatory arthritis, routine US use in newly referred patients seems to be associated with significantly earlier diagnosis and DMARD initiation.

**Electronic supplementary material:**

The online version of this article (10.1186/s12891-017-1850-4) contains supplementary material, which is available to authorized users.

## Background

It is widely accepted that early detection of persistent synovitis and initiation of disease-modifying anti-rheumatic drugs (DMARDS) in patients with Rheumatoid Arthritis (RA) is of critical importance [1, 2]. Assessment and initiation of DMARD therapy in RA at an early juncture has beneficial effects on both long-term clinical outcomes for patients and socioeconomic benefits [3, 4]. However there is still need to clarify the role of US imaging in the assessment of patients within early arthritis clinics as suggested by the 2016 update of the EULAR recommendations for the management of early arthritis [5].

Ultrasonography and MRI have consistently been shown to be more sensitive than clinical examination in detecting synovitis and predicting progression to persistent arthritis or RA [6, 7]. Previous studies have demonstrated that the use of ultrasound (US) improves diagnostic certainty in new patients presenting with seronegative early arthritis [8]. Additionally, US imaging has been consistently proven to be superior to plain radiographs in detecting erosions in the setting of early inflammatory arthritis [9, 10].

The aim of this study was to describe the impact of rheumatologist-performed US on the diagnosis and management of patients with early inflammatory arthritis in routine clinical practice. Our objectives were to compare 1) the time from first visit to treatment initiation (DMARDS) and 2) the time from first visit to formal diagnosis between patients with and without rheumatologist-performed US assessment; both overall and in a sub-population of patients with a final diagnosis of RA.

## Methods

This multi-centre prospective observational study was undertaken in four UK secondary/tertiary care rheumatology clinics (London [2 sites], Antrim and Southampton). All centres included a consultant who routinely used US at initial presentation to early arthritis clinics and at least one who did not, allowing comparison of decision making with respect to diagnosis and management.

### Participants

Patient aged ≥ 18 years at the time of presentation to the clinic and presenting with suspected new onset of inflammatory arthritis based upon the referral letter were recruited. At each participating centre, consultants received referrals form a similar pool of patients. All patients were unselected and were a true representation of the clinical workload undertaken in the study period.

All patients being referred to the early arthritis clinic were approached for recruitment. Patients were reviewed in consultant clinics where; 1) diagnosis and management decisions were routinely made with the use of rheumatologist-performed US (*US group*) **or** 2) diagnosis and management decisions were routinely made without the use of US (*non-US group*). Patients were randomly allocated into these groups without selection bias. Some patients had received investigations by their primary care physician prior to attending the Rheumatology service and these have been documented in Table 1.

**Table 1:** Patient demographics and sample characteristics at baseline, including tests carried out prior to initial clinic visit by referring primary physician

	US	Non-US	RA US	RA Non-US
Total no. Patients	134	124	56	58
Mean (standard deviation) age (years) at initial clinic visit	51.28 (15.75)	53.12 (17.34)	54.42 (17.21)	54.19 (17.75)
N (%)Male	42 (31%)	43 (35%)	17 (30%)	14 (24%)
N (%) Female	92 (69%)	81 (65%)	39 (70%)	44 (76%)
Median (IQR) time (months) from onset of symptoms to first clinic visit	5.98 (3.66 to 14.26)	5.26 (2.89 to 7.62)	5.36 (3.61 to 12.87)	4.78 (3 to 10.56)
Tests carried out prior to initial clinic visit by referring GP^a^				
N (%) Rheumatoid Factor	82 (61%)	81 (65%)	42 (75%)	45 (78%)
N (%) Anti-CCP	8 (6%)	9 (7%)	4 (7%)	6 (10%)
N (%) CRP	90 (67%)	78 (63%)	42 (75%)	36 (62%)
N (%) ESR	92 (69%)	84 (68%)	40 (71%)	41 (71%)
N (%) FBC	98 (73%)	74 (60%)	41 (73%)	40 (69%)
N (%) Joint x-ray (any joint)	39 (29%)	37 (30%)	17 (30%)	18 (31%)
ANA	51 (38%)	54 (44%)	26 (46%)	28 (48%)
Other^b^	67 (50%)	60 (48%)	27 (48%)	28 (48%)

^a^Abbreviations: anti-CCP = anti-cyclic citrullinated peptides; CRP = C-Reactive Protein; ESR = Erythrocyte sedimentation rate; FBC = full blood count; ANA = antinuclear antibodies

^b^Most commonly liver function, renal function and bone profile

### Ethical approval

Research ethics committee approval was obtained from the East London REC 2 (reference 10/H0704/25) prior to commencing the study. The study was carried out according to the principles of Good Clinical Practice. All participants provided written informed consent.

### Data collection

Data were collected from the initial clinic visit and three further subsequent visits, or until 1 year after the initial clinic visit. An independent *data collector* collected data from the medical notes of patients in both groups and the final locked database was provided to the statistician independent of any of the participating clinicians. The observation period varied depending on the timing of a patient’s clinic visits. For all patients diagnosed with RA, outcome data at one year after their initial clinic visit were also collected (sub-population). For patients in the US group, a physician questionnaire was used to evaluate the extent to which diagnosis and management decisions were affected by the results of their US scan (Additional file 1). All centres were blinded to the recruitment and diagnosis of patients within the Early Arthritis Clinics at other participating centres during the study period. Additional data on US technique, joints scanned and US findings were also collected. Investigations requested by primary care physicians, prior to attendance at an early arthritis clinic, was recorded.

### Statistical analysis

The sample size for the study was based on the historical frequency of patients with specific diagnosis and this informed the power of the study and sites selected. Analyses were stratified for the US and non-US groups. It was expected that there would be variation in the speed of diagnosis over time with an estimated initial 20% difference in diagnosis and treatment rates between each cohort. A sample size was submitted to the ethics committee with at least 100 patients in each arm providing sufficient power to demonstrate a difference in both the primary end point (time to treatment) and secondary end point (time to diagnosis).

Data for RA-diagnosed patients were analysed separately (sub-population). The non-parametric Mann-Whitney U test was used to test for significant differences between the US and non-US groups (both overall and for the RA-diagnosed subgroup) for time to diagnosis and time to treatment initiation (DMARDS). Only patients who received DMARDS during the follow up period were analysed for the time to treatment analysis. Fisher exact testing was used to compare percentages of patients diagnosed at their initial appointment and within one month.

## Results

A total of 258 patients were included in the study (134 US managed [range 7–55 per centre] and 124 non-US managed [range 6–65 per centre]).

The proportion of patients receiving a diagnosis of an inflammatory arthritis during the study period was 62% (83/134) in the US group and 65% (81/124) in the non-US group. 42% (56/134) of patients in the US group compared to 47% (58/124) in the non-US group were diagnosed with RA. Baseline patient characteristics are presented in Table 1 and a comprehensive list of diagnosis can be found in Table 2.

**Table 2:** Final diagnosis after 12 months of follow up by clinicians

Diagnosis	US	Non-US
Rheumatoid arthritis	56	58
Primary inflammatory arthritis (other than RA)	27	23
Mechanical or degenerative disorder	24	21
Connective tissue disease	4	3
Other systemic inflammatory disorder	2	4
Crystal arthritis	3	3
Metabolic disorder	2	3
Pain syndrome	4	2
Drug reaction	1	0
Not specified / unknown	11	7
Total	134	124

Median time between symptom onset and initial clinic visit in the US and non-US groups was 5.98 months and 5.26 months, respectively and 5.36 months and 4.78 months in the RA sub-population (Table 1).

Eleven patients were excluded from the US group and seven from the Non-US group subsequent analysis because of either a lack of data recorded or the referral to the early arthritis clinic was deemed inappropriate as symptoms and diagnosis had previously been established (Table 2). Analysis based on 123 patient in US and 117 in Non-US arm.

### All patients – US managed vs non-US managed groups

Both the median time to formal diagnosis and the time to treatment initiation (starting DMARDs) in the US group were less than in the non-US group: time to formal diagnosis 0.85 months versus 2.00 months (*p* = 0.0046); time to treatment initiation 0.62 months versus 1.41 months (*p* = 0.0048) (Table 3).

**Table 3:** Time (months) from initial clinic visit to diagnosis and treatment initiation (i.e. starting DMARDS)

	Time (months) to formal diagnosis				Time (months) to treatment initiation with DMARDS			
	US	Non-US	RA US	RA Non-US	US	Non-US	RA US	RA Non-US
Total	123	117	56	58	73	77	56	58
Mean	2.18	2.76	1.18	1.94	1.49	2.29	1.10	2.38
Median	0.85	2.00	0.23	1.38	0.62	1.41	0.46	1.81
SD	3.02	2.74	2.09	1.90	2.31	2.44	1.65	2.34
IQR	0.0 to 3.22	0.49 to 4.14	0.0 to 1.25	0.46 to 3.15	0.0 to 1.74	0.46 to 3.25	0.0 to 1.38	0.51 to 3.42
*P* value (Mann-Whitney U)	0.0046		0.0016		0.0048		0.0007	

In the US group 37% (45/123) of patients received a formal diagnosis at their initial clinic visit compared to 19% (22/117) in the non-US group (Fisher’s exact test *p* = 0.004); 54% (67/123) versus 32% (38/117) (US and non-US, respectively) received a formal diagnosis within one month of their initial clinic visit (*p* = 0.003). 60% (44/73) of US patients commenced treatment within 1 month of their initial clinic visit compared to 35% (27/77) of non-US (Fisher’s exact test, *p* = 0.006) as described in Table 3.

### RA diagnosed patient subgroup– US vs non-US managed groups

In the RA subpopulation the median time to formal diagnosis was 0.23 months and 1.38 months for the US and non-US groups, respectively (*p* = 0.016). The time to treatment initiation was also significantly lower in the US than in the non-US group (0.46 months versus 1.81 months, respectively, *p* = 0.0007) (Table 2).

**Fig. 1:**
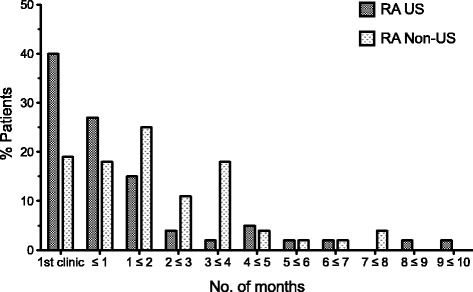
Distribution of time (months) from initial clinic visit to formal diagnosis – RA diagnosed patients only. Median time to formal diagnosis was 0.23 months and 1.38 months for the US and non-US groups, respectively (*p* = 0.014). 66% of US patients were diagnosed with in month (41% at their 1st clinic visit) compared to 36% of non-US patients (19% at their 1st clinic visit)

**Fig. 2:**
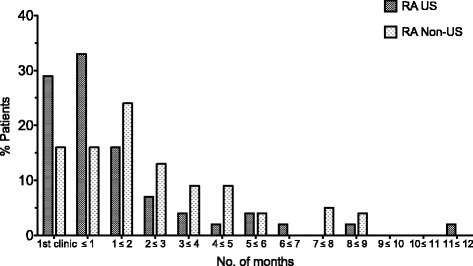
Distribution of time (months) from initial clinic visit to treatment initiation (DMARDS) – RA diagnosed patients only. Median time to treatment initiation was also significantly lower in the US than in the non-US group (0.46 months versus 1.81 months, respectively, *p* = 0.003). 61% of US cohort was treated within a month versus 31% of non-US cohort

For RA-diagnosed patients, a significantly greater proportion of patients in the US group than in the non-US group, 41% (23/56) versus 19% (11/58), received a formal diagnosis at their initial clinic visit (Fisher’s exact test *p* = 0.01) (Fig. 1). 66% (37/56) and 36% (21/58) of patients (respectively) received a formal diagnosis within one month of their initial clinic visit (*p* < 0.001) (Fig. 1). 61% (34/56) in the US group initiated treatment within one month of their initial clinic visit compared to 31% (18/58) in the non-US group (*p* = 0.002) (Fig. 2).

#### Physician US questionnaires

Physicians completed 162 ultrasound scan questionnaires in respect of 162 patient visits: 1st US scans (*n* = 120); 2nd US scans (*n* = 28); 3rd US scans (*n* = 12); and 4th US scans (*n* = 2). The majority of scans were used to aid diagnosis (93% of 1st scans and 75% of subsequent scans).

The joints most commonly scanned with US were the MCP and wrist joints (> 70% of US scans); the PIP joints (50–60% of US scans) and the IP joints (40–50% of US scans), with one centre routinely scanning all hand and wrist joints (Table 4). Joints scanned less frequently (< 10% of US scans) included the elbow, shoulder, knee, ankle and MTP joints. Mean (SD) number of joints scanned: all scans 13.5 (8.8), RA scans 12.1 (9.2) and first scans 16.1 (7.8). More than 20 joints were scanned at 55% of 1st scans.

For 69 US scans (43% of the 159 with data recorded) it was recorded by the physician that the US scan result had made a difference to the patient’s diagnosis when performed. The joints scanned and abnormalities detected in these cases (in terms of synovial thickening (ST) and positive power doppler (PD) signal) are shown in Table 5.

**Table 4:** Ultrasound data of all imaged joints. Data for 162 US scans were collected and frequency of scanning was calculated for each joint. One centre routinely scanned all wrist and hand joints. A high percentage of small joints of the hands with the right MCP 2 and both wrist joints being most frequently assessed

Joint^a^	LEFT SIDE (*n* = 162)	RIGHT SIDE (*n* = 162)
**MCP 1**	99 (61%)	102 (63%)
**MCP 2**	112 (69%)	122 (75%)
**MCP 3**	108 (67%)	117 (72%)
**MCP 4**	104 (64%)	110 (68%)
**MCP 5**	104 (64%)	114 (70%)
**IP**	70 (43%)	68 (42%)
**PIP 2**	88 (54%)	90 (56%)
**PIP 3**	87 (54%)	89 (55%)
**PIP 4**	74 (46%)	76 (47%)
**PIP 5**	74 (46%)	75 (46%)
**Wrist**	118 (73%)	121 (75%)
**Elbow**	5 (3%)	7 (4%)
**Shoulder**	2 (1%)	1 (1%)
**Knee**	10 (6%)	8 (5%)
**Ankle**	6 (4%)	6 (4%)
**Mid-foot**	1 (1%)	1 (1%)
**MTPs**	9 (6%)	12 (7%)
**Other** ^b^	12 (7%)	

^a^Abbreviations: MCP = metacarpophalangeal; IP = interphalangeal; PIP = proximal interphalangeal; MTP = metatarsophalangeal

^b^Other joints recorded as: flexor tendon, post. Tibial, hip, epicondyle, carpometacarpal, distal interphalangeal, toe

**Table 5:** Abnormalities detected at US scans reported as making a difference to diagnosis at that clinic visit. US abnormalities were commonly found at both sets of MCPs, PIPs and wrists. Lower limb joints are under represented in the ultrasound data set

Joint^a^	No. (%) joint scanned (*n* = 68)^b^	No. (%)^c^ with US abnormality	No. (%) ST only	No. (%) ST + PD
MCP1 L	47 (69.1%)	6 (12.8%)	5 (10.6%)	1 (2.1%)
MCP2 L	53 (77.9%)	19 (35.8%)	6 (11.3%)	13 (24.5%)
MCP3 L	51 (75.0%)	29 (56.9%)	17 (33.3%)	12 (23.5%)
MCP4 L	49 (72.1%)	21 (42.9%)	16 (32.7%)	5 (10.2%)
MCP5 L	50 (73.5%)	8 (16.0%)	1 (2.0%)	7 (14.0%)
MCP1 R	47 (69.1%)	3 (6.4%)	3 (6.4%)	0 (0.0%)
MCP2 R	52 (76.5%)	16 (30.8%)	2 (3.8%)	14 (26.9%)
MCP3 R	53 (77.9%)	20 (37.7%)	9 (17.0%)	11 (20.8%)
MCP4 R	48 (70.6%)	16 (33.3%)	9 (18.8%)	7 (14.6%)
MCP5 R	48 (70.6%)	11 (22.9%)	1 (2.1%)	10 (20.8%)
IP L	31 (45.6%)	1 (3.2%)	0 (0.0%)	1 (3.2%)
PIP2 L	40 (58.8%)	9 (22.5%)	2 (5.0%)	7 (17.5%)
PIP3 L	39 (57.4%)	10 (25.6%)	4 (10.3%)	6 (15.4%)
PIP4 L	37 (54.4%)	9 (24.3%)	3 (8.1%)	6 (16.2%)
PIP5 L	36 (52.9%)	9 (25.0%)	3 (8.3%)	6 (16.7%)
IP R	33 (48.5%)	1 (3.0%)	0 (0.0%)	1 (3.0%)
PIP2 R	39 (57.4%)	10 (25.6%)	4 (10.3%)	6 (15.4%)
PIP3 R	39 (57.4%)	15 (38.5%)	9 (23.1%)	6 (15.4%)
PIP4 R	35 (51.5%)	8 (22.9%)	3 (8.6%)	5 (14.3%)
PIP5 R	36 (52.9%)	8 (22.2%)	3 (8.3%)	5 (13.9%)
Wrist L	52 (76.5%)	18 (34.6%)	3 (5.8%)	15 (28.8%)
Wrist R	49 (72.1%)	19 (38.8%)	7 (14.3%)	12 (24.5%)
MTPs L	5 (7.4%)	5 (100.0%)	3 (60.0%)	2 (40.0%)
MTPs R	7 (10.3%)	7 (100.0%)	5 (71.4%)	2 (28.6%)

^a^Results presented for joints scanned at ≥ 5 US scans

^b^n = 68 (one questionnaire not included due to incomplete US findings)

^c^% of US scans at which each joint was imaged (e.g. for MCP1 (left) abnormalities were detected at 6 (12.8%) of 47 US scans)

## Discussion

### Main findings

This study provides real life data from four different UK Rheumatology centres on the impact of rheumatologist-performed US on the diagnosis and management of patients with inflammatory arthritis.

Data were collected for 258 patients; 134 had been referred to rheumatologists who routinely use US to aid diagnosis and management and 124 to rheumatologists who do not. Patients in both groups were similar in age (mean (SD) 51.3 (15.8) vs 53.1 (17.3) years in the US and non-US groups respectively) and gender distribution (69% and 65% females). In addition there was no significant difference in disease duration prior to presentation, inflammatory markers and clinical assessments as outlined in Table 1. The proportion of patients finally receiving a diagnosis of RA was not significantly different between populations at 12 months. The predominance of female patients and age of symptom onset are in line with previous literature reports [11, 12].

The importance of early detection of persistent synovitis and initiation of DMARDS in patients with RA is well documented [9, 13, 14]. Early diagnosis enables prompt initiation of disease modifying therapy, which can slow or halt disease progression and is associated with improved long-term functional and radiological outcomes. 92% of patients subsequently diagnosed clinically with RA fulfilled the ACR / EULAR 2010 criteria at 12 months. In this study, patients managed by rheumatologists who routinely used US to aid diagnosis and management received a formal diagnosis and were initiated on DMARDs significantly earlier than those managed by rheumatologists who did not use US routinely. These differences were significant both in the overall group (median time to diagnosis 0.85 months US vs 2.00 non-US; median time to treatment initiation 0.62 months US vs 1.41 non-US) and in the subpopulation of RA-diagnosed patients (median time to diagnosis 0.23 months US vs 1.38 non-US; median time to treatment initiation 0.46 months US vs 1.81 non-US). While the time to diagnosis encompasses both inflammatory and non-inflammatory conditions, the difference to time of formal diagnosis seems to translate to earlier initiation of DMARD therapy. The proportion of patients who initiated treatment within one month of first outpatient appointment was significantly higher in the US when compared to the US group (57% vs 35% overall; 62% vs 33% RA).

However, although there were statistically significant differences in time to diagnosis and treatment initiation in the US vs the non-US group, the clinical significance of a 6 week reduction in time to DMARD initiation (as observed in the RA subgroup in this study) in terms of impact on radiographic erosions, functional outcomes and economic burden, is unclear and requires further investigation. Moreover, the potential delays in referrals from primary care physicians is likely to presents a significant challenge to clinicians in achieving further reductions in time to treatment initiation.

In this study 162 prospective physician questionnaires were completed, providing valuable information about the current use of US in routine UK clinical practice. In most cases US scans were used to aid diagnosis (93% of 1st scans, 75% of all scans). The stated reason for US was to assess or monitor for sub-clinical disease (35%) in more cases than to inform treatment changes (7%). Physicians reported that in 43% of cases, US scans had made a difference to the diagnosis, indicating that US is linked to clinical decision making processes. The difference appears to be in the time to diagnosis rather than the diagnosis itself, since the proportion of patients diagnosed with RA was similar in the US and non-US groups (42% vs 47%, respectively).

In terms of the number and types of joints scanned; there was a tendency to scan more joints at 1st scans (more than 20 joints were scanned at 55% of 1st scans, for all scans this was 43%) with overall, a mean of 13.5 joints scanned at each US assessment. The MCPs (particularly 2nd and 3rd), wrist and PIP joints were heavily represented, both in terms of joints scanned and detection of abnormalities (for the US scans which were reported as having made a difference to diagnosis), with the 2nd and 3rd MCPs and wrists scanned at over 70% of all US scans. There is currently a lack of consensus about the joint regions and optimal or minimum number of joints, which should be targeted for routine US data set collection, and work is currently underway to develop a single standardised US scoring system that can be used in routine practice to reflect overall disease activity [15, 16]. Whilst a weakness of this study may be the lack of standardised data set scanning across sites, the primary focus of this work was to gather real world information regarding the practicalities of US imaging in early arthritis clinics and the feasibility of performing a wide data set collection. In the context of a prospective randomised trial a limited core data set would improve the study design. The relatively under imaged MTP joints may have provided additional information to clinicians improving the time to diagnosis and treatment in the US cohort. Recently published work has demonstrated that US evaluation of a specific core joint group could potentially be used to assess overall inflammatory activity [17]. Proposed scores include a 12 joint assessment score using the wrists, second and third MCP, second and third PIP of the hands and knee joints (Naredo et al.) [7] and the US7, a 7 joint score (Backhaus et al.) which uses the following joints of the clinically dominant hand and foot: wrist, second and third MCP and PIP and second and fifth MTP joints [18, 19]. Our study supports the inclusion of these joints in a core dataset with a high incidence of significant of ultrasonographic abnormalities detected and the incorporation of these findings into management decisions. The findings indicate that this approach is both feasible in real world clinical practice and yields good dividends where there is a reasonable index of suspicion.

### Limitations

This study was undertaken in only four secondary/tertiary care centres. Nevertheless, some significant differences between groups were observed, with important implications for clinical practice. Patients were recruited to this study in a prospective fashion in an unselected manner, however, data were collected retrospectively from patient notes and it was not possible to obtain any missing data items.

Different rheumatologists managed patients in each group. Therefore the results may have been affected by confounding factors related to other differences between the practices of the rheumatologists. However the effect of a single rheumatologist, or single site, was minimised by a multi-centre collaboration in 4 centres and a prospective unselected patient recruitment to the study.

## Conclusion

In this study of newly referred patients with inflammatory arthritis, use of US was associated with more rapid diagnosis of synovitis and earlier initiation of DMARDS; this is known to have beneficial effects on patient outcomes and its importance has been recognised by NICE, the National Audit office and the Department of Health in recent changes to commissioning guidance. Overall, the findings of the study support the use of US by rheumatologists at the bedside and reflect the growing interest in the use of US to assess joint inflammation.

## Additional files

Additional file 1: This file is the US questionnaire completed by Rheumatologists after using an US assessment in clinic. (DOC 77 kb)

## Abbreviations

DMARDS: Disease-modifying anti-rheumatic drugs
RA: Rheumatoid Arthritis
