# Supplementary material for: The impact of rheumatologist-performed ultrasound on diagnosis and management of inflammatory arthritis in routine clinical practice
Source: BMC Musculoskelet Disord. 2017 Nov 22;18:487. doi: 10.1186/s12891-017-1850-4 (PMC5700521; doi:10.1186/s12891-017-1850-4)
Supplement: Supplementary file 1 — This file is the US questionnaire completed by Rheumatologists after using an US assessment in clinic. (DOC 77 kb) [file 12891_2017_1850_MOESM1_ESM.doc]

| **Research study to describe how ultrasound is currently being used and the impact it has on the diagnosis and management pathways of patients with rheumatoid arthritis (RA)** |
| --- |

**Ultrasound patients only**

(Prospective data collection by rheumatologist – 1 sheet / ultrasound)

Provisional diagnosis before ultrasound:

Provisional management decision before ultrasound:

Date of ultrasound: / /

Primary reason for use of ultrasound: Diagnosis

Monitoring of inflammatory arthritis

Sub-clinical monitoring

Injection guiding

Other; specify:

Importance of factors in making diagnosis / management decision:

|  | **Not important** | **Minor importance** | **Medium importance** | **Very important** |
| --- | --- | --- | --- | --- |
| **DAS** |  |  |  |  |
| **HAQ** |  |  |  |  |
| **CRP** |  |  |  |  |
| **X-ray** |  |  |  |  |
| **US** |  |  |  |  |
| **MRI** |  |  |  |  |

Did ultrasound make a difference to diagnosis at this consultation: Yes No

Did ultrasound make a difference to management decision: Yes No

Was ultrasound used to assess sub-clinical disease: Yes No

Was ultrasound used to treat sub-clinical disease: Yes No

**Continued over ........**

Joints/tendons scanned and results:

*(Can be transcribed by researcher if this information is already recorded in the patients’ records)*  Synovial

Doppler Doppler Synovial thickening

Joint/tendon Side Type signal graded thickening graded Erosions Other

*scanned (L/R) (L/T) (Yes/No) (Yes*/No/NA) (Yes/No) (Yes*/No) (Yes/No) (Yes**/No)*

| MCP 1 | L |  |  |  |  |  |  |  |
| --- | --- | --- | --- | --- | --- | --- | --- | --- |
| MCP 2 | L |  |  |  |  |  |  |  |
| MCP 3 | L |  |  |  |  |  |  |  |
| MCP 4 | L |  |  |  |  |  |  |  |
| MCP 5 | L |  |  |  |  |  |  |  |
| MCP 1 | R |  |  |  |  |  |  |  |
| MCP 2 | R |  |  |  |  |  |  |  |
| MCP 3 | R |  |  |  |  |  |  |  |
| MCP 4 | R |  |  |  |  |  |  |  |
| MCP 5 | R |  |  |  |  |  |  |  |
| IP | L |  |  |  |  |  |  |  |
| PIP 2 | L |  |  |  |  |  |  |  |
| PIP 3 | L |  |  |  |  |  |  |  |
| PIP 4 | L |  |  |  |  |  |  |  |
| PIP 5 | L |  |  |  |  |  |  |  |
| IP | R |  |  |  |  |  |  |  |
| PIP 2 | R |  |  |  |  |  |  |  |
| PIP 3 | R |  |  |  |  |  |  |  |
| PIP 4 | R |  |  |  |  |  |  |  |
| PIP 5 | R |  |  |  |  |  |  |  |
| Wrist | L |  |  |  |  |  |  |  |
| Wrist | R |  |  |  |  |  |  |  |
| Elbow | L |  |  |  |  |  |  |  |
| Elbow | R |  |  |  |  |  |  |  |
| Shoulder | L |  |  |  |  |  |  |  |
| Shoulder | R |  |  |  |  |  |  |  |
| Knee | L |  |  |  |  |  |  |  |
| Knee | R |  |  |  |  |  |  |  |
| Ankle | L |  |  |  |  |  |  |  |
| Ankle | R |  |  |  |  |  |  |  |
| Mid-foot | L |  |  |  |  |  |  |  |
| Mid-foot | R |  |  |  |  |  |  |  |
| MTPs | L |  |  |  |  |  |  |  |
| MTPs | R |  |  |  |  |  |  |  |
|  |  |  |  |  |  |  |  |  |
|  |  |  |  |  |  |  |  |  |
|  |  |  |  |  |  |  |  |  |

* If yes record score

** If yes record
